# Antimalarials in Lupus Nephritis: How Strong Is the Evidence?

**DOI:** 10.34067/KID.0000000626

**Published:** 2024-10-21

**Authors:** Fernando Caravaca-Fontán, Federico Yandian, Ladan Zand, Sanjeev Sethi, Fernando C. Fervenza

**Affiliations:** 1 Department of Nephrology, Instituto de Investigación Hospital “12 de Octubre” (imas12), Madrid, Spain; 2 Department of Nephrology, Hospital de Clínicas “Dr. Manuel Quintela”, Montevideo, Uruguay; 3 Division of Nephrology and Hypertension, Mayo Clinic, Rochester, Minnesota; 4 Department of Laboratory Medicine and Pathology, Mayo Clinic, Rochester, Minnesota

**Keywords:** clinical nephrology, immunosuppression, lupus nephritis, mycophenolate mofetil, SLE

## Abstract

SLE is a chronic multisystem autoimmune disease that affects the kidneys in approximately 50% of patients, with the prevalence rising to as high as 70% in certain populations, such as African American and Asian people. Antimalarials—and particularly hydroxychloroquine (HCQ)—are currently considered a mainstay of therapy, together with immunosuppressants. Over the past decades, several studies have extensively investigated the mechanisms of action of antimalarial agents and their potential beneficial properties in patients with SLE in general. However, the evidence for the therapeutic benefit of HCQ in patients with lupus nephritis (LN) derives mainly from observational studies, conducted in an era before the refinement of induction and maintenance protocols for immunosuppressive therapy. Despite the paucity of high-quality evidence on its efficacy in LN, the nephrology community widely supports the universal use of HCQ in patients with LN, and recommendations for its use are firmly entrenched in various clinical practice guidelines. Nonetheless, the use of antimalarials may also carry inherent risks, underscoring the importance of personalized approaches in these patients. Herein, we comprehensively review the available literature on antimalarials in LN, aiming to update the current evidence, limitations, and future perspectives for the use of antimalarials in adults.

## Introduction

SLE affects the kidneys as lupus nephritis (LN) in approximately 50% of patients, with the prevalence rising to as high as 70% in certain populations, such as African American and Asian people.^1,2^ Antimalarials (*e*.*g*., chloroquine and hydroxychloroquine [HCQ]) are among the oldest drugs used in SLE and are considered a mainstay of therapy, together with immunosuppression (IS).^3,4^

HCQ is a lipophilic 4-aminoquinoline drug, with large volume of distribution, accumulating in acidic compartments, like lysosomes.^5^ Its long half-life (40–60 days) means steady-state levels are reached after about 6 months.^4^ HCQ levels vary between patients, complicating adherence and efficacy assessment, because blood level measurements are technically challenging.^5–7^ It is metabolized *via* cytochrome P450 enzymes, leading to potential drug interactions, and excreted by the kidneys.^5^

The potential beneficial properties of antimalarials in SLE include^8–12^ interference with T-cell antigen presentation, inhibition of lysosome-dependent autophagy, reduction of cytokine production, and antiproliferative effects.

Clinical practice guidelines for LN (European League for Rheumatism jointly with the European Renal Association, American College of Rheumatology guidelines and Kidney Disease Improving Global Outcomes) recommend the use of HCQ for all patients, unless contraindicated.^13–16^

These recommendations are based primarily on studies demonstrating efficacy of HCQ in non-LN.^3,17–23^ However, there is a paucity of high-quality evidence in patients with LN receiving contemporary standard of care (SoC) treatment, which includes maintenance of IS after clinical remission is achieved.

Furthermore, evidence arises mostly from observational studies that predate the era of induction of remission with dual IS (*e*.*g*., corticosteroids [CS]±cyclophosphamide [CyC] or mycophenolate mofetil [MMF]).^13,15,24^ In our view, the definition of remission used in many of these studies—based solely on proteinuria, inactive urinary sediment, and stable kidney function—is incomplete because it overlooks the presence or absence of immunological remission (*i*.*e*., normal complement levels and negative anti-double stranded DNA antibodies [anti-dsDNA]).^25,26^ Although this concept has yet to be fully demonstrated in SLE and LN, it has been successfully applied in other autoimmune diseases,^27–30^ providing a rationale for its potential extrapolation to patients with LN. The role of immunological remission in LN has been recently reviewed.^32^

Therefore, this raises speculation as to whether antimalarial treatment could provide significant additional benefits to all patients with active LN undergoing the current SoC or whether a personalized prescribing approach based on patients' underlying characteristics or extrarenal manifestations should be considered instead.

We hereby comprehensively assess the strength of the evidence supporting the use of HCQ in patients with LN, highlighting both the strengths and limitations of the studies.

## Effect of HCQ on Remission of LN or Kidney Function Recovery

The association between HCQ use and likelihood of remission in LN was assessed in several studies (Table 1).

**Table 1:** Summary of studies that assessed the association between HCQ use and LN remission or recovery of kidney function

Study/Year	Design	Population	Treatments/Intervention	Outcomes
Barber *et al.*^32^ 2006	Retrospective study	35 patients with LN (mean age 37±2 yr)	CS: 33 (94%) Aza: 12 (34%) CyC: 19 (54%) PEX: 5 (14%) MTX: 1 (3%) HCQ: 25 (71%)	16 (46%) achieved SR versus 19 (54%) in a mean 38±7 mo Patients with remission were more likely to have been treated with HCQ (94% versus 53% in controls) Multivariate analyses: female sex, older age, higher nonrenal SLEDAI and absence of Aza were predictors of SR
Kasitanon *et al.*^65^ 2006	Cohort study	29 patients with membranous LN with mean eGFR 126±54 ml/min per 1.73 m^2^ (mean age 30±12 yr)	MMF: 29 (100%) HCQ: 11 (38%)	11 (38%) achieved CR by 12 mo 7/11 (64%) taking HCQ achieved remission by 12 mo, compared with 4/18 (22%) not on HCQ (*P* = 0.036) Association persisted after controlling for anti-dsDNA antibody
Shinjo *et al.*^33^ 2009	Retrospective study	57 patients with SLE and ≥65 yr. 6 (11%) with renal involvement	CQ: 38 (67%)	43 (75%) patients achieved disease remission and 14 (25%) with disease activity Disease remission was strongly associated with antimalarial therapy (OR, 12.91; 95% CI, 2.87 to 58.13)
Shaharir *et al.*^66^ 2014	Retrospective study	150 patients 89% of which had biopsy-proven LN Mean age 36±11 yr	HCQ: 90 (60%) CS: 150 (100%) Aza: 85 (57%) CsA: 71 (47%) MMF: 78 (52%) CyC: 117 (78%)	Patients who received early HCQ (≤3 mo after diagnosis), optimum HCQ dose at 6.5 mg/kg per day and achieved early CR were less likely to have disease damage (*P* < 0.05)
Lee *et al.*^34^ 2020	Retrospective longitudinal cohort study	90 patients with LN with mean eGFR 37±14 ml/min per 1.73 m^2^ (mean age 38±13 yr)	HCQ: 29 (32%) CS: 88 (98%) CyC: 66 (73%) MMF: 10 (11)	46 (51%) recovered kidney function after 6 mo On multivariate analysis, HCQ (OR, 3.89; 95% CI, 1.19 to 12.65; *P* = 0.024), prolonged LN (OR, 0.93; 95% CI, 0.87 to 0.98; *P* = 0.009), and high-grade tubular atrophy (OR, 0.45; 95% CI, 0.21 to 0.83; *P* = 0.013) were associated with renal function recovery
Gheet *et al.*^35^ 2023	Double-blind, randomized, placebo-controlled trial	60 children with proliferative LN treated with CS+MMF	HCQ group (*n*=30) Placebo group (*n*=30)	After 12 mo, triglycerides, cholesterol, proteinuria, anti-dsDNA, and SLEDAI score were significantly decreased in the HCQ group (*P*: 0.002, 0.012, 0.031, 0.001, respectively) The cumulative probabilities of developing partial and CR were 40% and 60% in the HCQ group versus 53.3% and 36.7% in placebo group (*P* = 0.002) HCQ group experienced mucocutaneous alopecia (3.3%), hyperpigmentation (10%), and ophthalmological mild retinal changes (6.7%), but they did not differ significantly from the placebo group

Anti-dsDNA, anti-double stranded DNA antibodies; Aza, azathioprine; CI, confidence interval; CQ, chloroquine; CR, complete remission; CS, corticosteroids; CsA, cyclosporine; CyC, cyclophosphamide; HCQ, hydroxychloroquine; LN, lupus nephritis; MMF, mycophenolate mofetil; MTX, methotrexate; OR, odds ratio; PEX, plasma exchange; SLEDAI, systemic lupus erythematosus disease activity index; SR, sustained remission.

Barber *et al*.^32^ evaluated predictors of sustained remission (SR), defined as normal kidney function, proteinuria <0.5 g/d, inactive urinary sediment, and minimal IS (prednisone <10 mg/d and antimalarials). Patients achieving SR were more frequently treated with HCQ (94% versus 53%), although HCQ was not included in the SR predictor analysis. Moreover, although CS were used in 94% of patients, CyC and azathioprine (Aza) were less commonly administered (54% and 34%, respectively), and no maintenance therapy was instituted. Thus, patients in this study were not treated according to the current SoC.

Shinjo *et al*.^33^ found that disease remission was strongly associated with antimalarial therapy (odds ratio [OR], 12.91; 95% confidence interval [CI], 2.87 to 58.13) in 57 patients with SLE age ≥65 years. However, only 11% had kidney involvement, and the study was from 1970 to 1980, before current SoC. Treatment allocation was physician-dependent, and the author acknowledged that the observed benefits of antimalarials could be due to confounding.

Lee *et al*.^34^ analyzed kidney function recovery at 6 months (eGFR ≥60 ml/min per 1.73 m^2^) in 90 patients with class 3/4±5 LN and reduced eGFR (mean 37±14 ml/min per 1.73 m^2^). After IS (CS, 98%; CyC, 73%; MMF, 11%), 51% recovered. HCQ was used in 32% of cases. Multivariable analysis showed that HCQ, prolonged LN, and tubular atrophy were linked to recovery. As acknowledged by the authors, limitations included indication bias as HCQ use was discretionary, 11% received no IS, and other factors affecting kidney impairment were not considered.^34^

In summary, the aforementioned studies suggesting HCQ's effect on LN remission were small, retrospective, and prone to bias. One might speculate that patients perceived by physicians as having an intrinsically better prognosis may have been more likely to receive HCQ, introducing a potential bias by indication.

A recent study by Gheet *et al*.^35^ in Egypt evaluated the efficacy and side effects of HCQ in children with proliferative LN through a double-blind, randomized, placebo-controlled trial involving 60 children treated with steroids and MMF. Patients were divided into two groups: HCQ (*n*=30) and placebo (*n*=30). At 12 months, the HCQ group showed significant reductions in lipid profiles, proteinuria, anti-dsDNA levels, and systemic lupus erythematosus disease activity index scores. The cumulative probabilities of achieving partial and complete remission were 40% and 60% in the HCQ group compared with 53.3% and 36.7% in the placebo group (*P* = 0.002), respectively. Although this well-designed study revealed that adjunctive treatment with HCQ led to improved kidney outcomes, it had important limitations, including a small pediatric sample size, short follow-up duration, and lack of serum HCQ levels. In addition, there were no details on per-protocol analyses or actual immunosuppressive doses in each treatment arm. Baseline proteinuria was numerically higher in the placebo group (2.8 versus 2.2 g/d; *P* = 0.11), which could have influenced the results because patients with higher initial proteinuria often require more time to achieve remission. No similar studies have been conducted in adults and results of the study have not been confirmed by other investigators.

## Effect of HCQ on LN Flares

Flares remain a major clinical concern,^8^ driving recommendations for the use of maintenance therapy. Several studies have evaluated antimalarials' potential benefits in sustaining remission (Table 2).

**Table 2:** Summary of studies that assessed the association between HCQ and LN flares

Study/Year	Design	Population	Treatments/Intervention	Outcomes
Tsakonas *et al.*^37^ 1998	Randomized withdrawal trial	47 patients with quiescent SLE	Randomized to HCQ (*n*=25) or placebo (*n*=22) over 42 mo of study	11/22 (50%) patients randomized to placebo and 7/25 (28%) randomized to HCQ experienced a major flare (nonsignificant RR, 0.43; 95% CI, 0.17 to 1.12) RR for LN flare: 0.26 (95% CI, 0.17 to 2.41)
Moroni *et al.*^38^ 2013	Retrospective study	161 patients with LN who achieved a stable clinical remission (normal SCr, proteinuria <0.5 g/d, inactive urinary sediment and no extra-renal manifestations of SLE)	Attempt to slowly and progressively eliminate steroids and immunosuppressive drugs in 73 (45%) patients	21/73 patients (29%) developed flares during treatment tapering, which required reinforced treatment 20/21 patients (95%) with relapses entered remission again; 1/21 (5%) achieved partial remission 52/73 patients (71%) treatment was interrupted. Of them, 20 patients (38%) had subsequent flares Patients without flares had received treatment for longer period, had longer remission, and treatment with CQ was continued Patients in which treatment was successfully interrupted had lower kidney failure, HTN, and cardiovascular events
Fasano *et al.*^39^ 2021	Retrospective study	154 patients with SLE at remission	56 (36%) CS withdrawal and 98 (64%) continuous CS dose (5 mg) for 1 yr HCQ in 71% of CS withdrawal group versus 71% in continuous CS dose	17 flares during a median follow-up of 59 mo, similar between groups On multivariate analysis, long-term HCQ treatment (HR, 0.84; *P* = 0.03) and 5-yr lasting remission at CS withdrawal (HR, 0.12; *P* = 0.0003) were found to be protective, whereas SACQ disease (HR, 22.88; *P* = 0.003) and history of LN (HR, 3.38; *P* = 0.01) increased the risk
Zen *et al.*^40^ 2022	Retrospective analysis of prospectively collected data	238 patients with LN treated with IS (mean age 47±14 yr, mean disease duration 18±10 yr)	Treatment with MMF (77%), Aza (46%), CyC (40%), CsA (17%), Tac (6%) 83 (35%) discontinued IS after remission	During a follow-up of 116.5±78 mo, 19 patients (22.8%) developed a flare (8/19 renal) and were retreated; 14/19 (73.7%) reachieved remission after restarting therapy On multivariable analysis, antimalarial therapy (OR, 0.19; 95% CI, 0.04 to 0.98; *P* = 0.047), age at IS discontinuation (OR, 0.93; 95% CI, 0.87 to 0.99; *P* = 0.04), and remission duration >3 yr before IS discontinuation (OR, 0.23; 95% CI, 0.06 to 0.92; *P* = 0.038) were protective against disease flares
Gomez *et al.*^44^ 2023	Pooled data from BLISS-52, BLISS-76, BLISS-SC and BLISS-Northeast Asia randomized controlled trials	3225 patients with active SLE yet no severe ongoing LN	Intravenous belimumab 1 mg/kg; intravenous belimumab 10 mg/kg; subcutaneous belimumab 200 mg; or placebo in addition to standard therapy	192 patients developed a renal flare after a median of 197 d 2173 (67.3%) patients were on antimalarials Use of antimalarials yielded a lower risk of renal flares (HR, 0.66; 95% CI, 0.55 to 0.78; *P* < 0.001) The protection conferred was enhanced when belimumab and antimalarials were coadministered
You *et al.*^45^ 2024	Multicenter, observer-blinded randomized clinical trial	130 treatment-naive patients with newly diagnosed SLE, a high titer of anti-dsDNA antibody, and no major organ involvement	Randomized 1:1 to oral CS (0.5 mg/kg per day) and HCQ (5 mg/kg per day) (control group) or CS (0.5 mg/kg per day) and HCQ (5 mg/kg per day) plus MMF (500 mg twice daily) (MMF group) for 96 wk	The risk of severe flare was significantly lower in the MMF group (10.8%) versus the control group (27.7%) (RR, 0.39 [95% CI, 0.17 to 0.87]; *P* = 0.01) One of 65 patients in the MMF group (1.5%) and nine of 65 in the control group (13.8%) manifested LN (RR, 0.11 [95% CI, 0.01 to 0.85]; *P* = 0.008)

Anti-dsDNA, anti-double stranded DNA; Aza, azathioprine; CI, confidence interval; CQ, chloroquine; CS, corticosteroids; CsA, cyclosporine; CyC, cyclophosphamide; HCQ, hydroxychloroquine; HR, hazard ratio; HTN, hypertension; IS, immunosuppression; LN, lupus nephritis; MMF, mycophenolate mofetil; MTX, methotrexate; OR, odds ratio; RR, relative risk; SACQ, serologically active clinically quiescent; SCr, serum creatinine; Tac, tacrolimus.

The Canadian HCQ Study Group assessed HCQ's effectiveness in preventing flares in patients with quiescent SLE.^36^ In a 24-week double-blind withdrawal trial, 47 patients were randomized to continue HCQ (*n*=25) or placebo (*n*=22). At the end of 24 weeks, clinical flare-ups occurred in 16 patients on placebo (73%) and nine on HCQ (36%).^36^ As part of a trial extension, patients were evaluated for an additional 3 years.^37^ Eleven patients (50%) randomized to placebo and 7 (28%) randomized to HCQ experienced a major flare, yielding a nonsignificant trend in the relative risk (RR) of major flare in those assigned to treatment (0.43; 95% CI, 0.17 to 1.12). Notably, despite the absence of maintenance IS, only four kidney flares occurred: one in the HCQ group and three in the placebo group (RR, 0.26; 95% CI, 0.03 to 2.54; *P* = 0.25). Thus, this study did not demonstrate HCQ's benefit in preventing major LN flares in patients without maintenance therapy.^37^ In addition, no data on anti-dsDNA and C3 or C4 levels at randomization were provided.

In a retrospective study by Moroni *et al*.,^38^ 73 of 161 patients with LN attempted to interrupt IS after achieving clinical remission, defined as normal serum creatinine, proteinuria <0.5 g/d, and no extrarenal manifestations. Twenty-one patients (29%) developed flares during withdrawal and were retreated. The remaining 52 patients (71.2%) completely withdrew treatment; 32 (group A) did not resume therapy (median follow-up 101.8 months), whereas 20 (group B) experienced at least one flare (median follow-up 37 months) and were retreated. After a median follow-up of 286 months, ten of the 20 patients were off therapy. Patients who never relapsed had received longer IS (98.1 versus 31.0 months) and experienced longer remission (52.8 versus 12.0 months) before discontinuation. They continued HCQ after stopping IS, suggesting a potential benefit of HCQ. However, some patients had ongoing immunological activity, indicated by low C3/C4 and positive anti-dsDNA. In addition, patients in group A exhibited lower proteinuria and higher use of cytotoxic drugs compared with group B. Consequently, the lower relapse risk of relapse appears to find a more plausible explanation in the context of milder disease and effective therapies rather than being solely attributed to HCQ.

Another recent retrospective study^39^ evaluated predictors of flares in 154 patients with SLE in remission, 54% with a history of LN. Of them, 36% had CS discontinued, whereas 64% continued low-dose CS; 71% in both groups remained on HCQ. Overall, 11% experienced flares, with no significant differences between groups (11% versus 12%). A longer duration of HCQ treatment and remission (>5 years) were protective factors. The study introduced the concept of serologically active clinically quiescent disease, revealing a higher flare rate in serologically active clinically quiescent patients (54%) versus serologically inactive patients (2%; *P* < 0.0001). Anti-dsDNA and reduced complement levels were strong predictors of lupus flares.

Zen *et al*.^40^ reported similar findings in a retrospective study involving patients with LN. Eighty-three patients (35%) in clinical or complete clinical and serological remission underwent gradual IS discontinuation after a median remission period of 46 months. During a mean follow-up of 10 years, 23% developed a flare, with renal flares occurring in 9.6% of patients. Most patients continued treatment with HCQ (81.9%), whereas 10.8% received low-dose glucocorticoids and 7.2% discontinued all treatment. Flares were significantly less frequent in those treated with HCQ (16.2%) compared with those without (53.3%; OR, 0.145; 95% CI, 0.039 to 0.536; *P* = 0.002). Multivariable analysis indicated that antimalarial therapy (OR, 0.194; *P* = 0.047), age at IS withdrawal (OR, 0.93; *P* = 0.040), and remission duration >3 years (OR, 0.231; *P* = 0.038) were protective against disease flares, although kidney flares were not specifically analyzed because of the small number of patients. Notably, all severe flares occurred in patients with serological activity, highlighting that clinical remission alone may not accurately reflect autoimmune activity. Anti-dsDNA antibodies were associated with kidney disease, suggesting they can identify patients at higher risk of flare. Thus, discontinuing maintenance IS in patients with ongoing serological activity is not recommended.

In a *post hoc* analysis of the Aspreva Lupus Management Study,^41^ Dall’Era *et al*.^42^ evaluated the predictive value of various demographic, clinical, laboratory, and histopathologic features on the rates of complete remission and treatment failure (TF) during the maintenance phase of the trial involving 370 patients with class 3–5 LN. Factors independently associated with a higher likelihood of TF included baseline anti-dsDNA positivity, failure to reduce anti-dsDNA levels within 8 weeks, and failure to decrease the urinary protein-to-creatinine ratio by ≥25% in the same timeframe, but not the use of HCQ. Among the 227 patients entering the maintenance phase, 116 were randomized to receive MMF and 111 to Aza,^43^ with antimalarials used by 38.3% of MMF and 36.9% of Aza patients. Factors associated with TF included lack of antimalarial treatment, failure to decrease anti-dsDNA and urinary protein-to-creatinine ratio during the initial 8 weeks, and anti-dsDNA positivity at the end of induction.^42^ However, only 37% of patients were taking antimalarials, with a modest effect observed: 26% of patients not treated with antimalarials experienced TF versus 21% of treated patients (OR, 2.4; 95% CI, 1.1 to 5.1; *P* = 0.02). Notably, the multivariate analysis did not account for the differing treatments (MMF versus Aza), leaving the confounding impact of MMF use unaddressed.

A recent large study pooled data from four randomized control trials (BLISS-52, BLISS-76, BLISS-SC, and BLISS-Northeast Asia, *N*=3225) to evaluate the impact of antimalarials and various doses of belimumab on preventing renal flares.^44^ In total, 192 patients experienced a renal flare after a median duration of 197 days, with 2173 patients (67%) receiving antimalarial treatment. The incidence of renal flares was significantly lower among patients treated with antimalarials (56 per 1000 person-years) compared with those without (78 per 1000 person-years), with regression analyses indicating a reduced risk of renal flares (hazard ratio, 0.66; 95% CI, 0.55 to 0.78; *P* < 0.001). The protective effect was further enhanced when belimumab and antimalarials were coadministered, showing the lowest flare rate for intravenous belimumab 1 mg/kg combined with antimalarials (18.5 cases per 1000 person-years).^44^ This study exhibits notable strengths in its extensive patient cohort and the consistency of its data.

Key limitations included the lack of blood concentration data for antimalarials and the absence of patients with severe active LN, which could affect generalizability. The authors noted that exposure to HCQ was based on prescriptions during a 30-day period before trial initiation, highlighting a risk of misclassification because of common nonadherence. Interestingly, the most significant benefit was observed with belimumab at 1 mg/kg, although the FDA-approved dosage is 10 mg/kg or 200 mg subcutaneously. The highest incidence of renal flares was noted in patients receiving 10 mg/kg belimumab, raising questions about the efficacy of higher doses. Furthermore, nearly a quarter of patients in the 1 mg/kg group were also on Aza, warranting further investigation. The study also lacked information regarding baseline characteristics of patients who experienced flares versus those who did not, raising concerns about potential imbalances in the study population.

Finally, a recent randomized controlled trial in Asian patients evaluated the efficacy of MMF combined with prednisone and HCQ in preventing SLE flares.^45^ The study randomized 130 patients to receive either the combination of MMF, prednisone, and HCQ or prednisone and HCQ alone over a 96-week period. The study revealed that MMF significantly reduced the rate of severe flares (RR, 0.39; 95% CI, 0.17 to 0.87; *P* = 0.01), unlike HCQ, and notably decreased the incidence of LN in patients with new-onset SLE and high titers of anti-dsDNA antibodies (RR, 0.11; 95% CI, 0.01 to 0.85; *P* = 0.008).^45^

Further prospective studies that compare patients in complete clinical and serological remission who are randomized to continue on maintenance IS versus HCQ alone are needed to elucidate the true efficacy of HCQ in preventing LN flares.^46^

## Effect of HCQ on Risk of Kidney Failure in LN

LN significantly affects morbidity and mortality in SLE, with 10%–30% of patients progressing to kidney failure.^1,2^ Several studies have examined antimalarials' efficacy in preventing this outcome (Table 3).

**Table 3:** Summary of studies that assessed the association between hydroxychloroquine and risk of kidney damage/failure

Study/Year	Design	Population	Treatments/Intervention	Outcomes
Sisó *et al.*^47^ 2008	Retrospective study	206 patients with LN (mean age 30 yr, range 8–67)	Induction therapy included CS (84%) and CyC (28%) Patients were classified as those who never received HCQ (*n*=150) and those with HCQ (*n*=56)	Patients exposed to HCQ had a lower frequency of creatinine values >4 mg/dl (2% versus 11%, *P* = 0.029) and kidney failure (2% versus 11%, *P* = 0.044) Patients exposed to HCQ had a lower frequency of HTN (32% versus 50%, *P* = 0.027), infections (11% versus 29%, *P* = 0.006), thrombotic events (5% versus 17%, *P* = 0.039), and lower mortality (2% versus 13%, *P* = 0.029)
Pons-Estel *et al.*^49^ 2009	Longitudinal observational cohort	203 patients of whom 63 (31%) developed kidney damage (eGFR <50%, 24 h-proteinuria ≥3.5 g/d, or kidney failure)	CS: 100% Aza: 31% CyC: 33% MMF: 8% HCQ: 79%	Patients who received HCQ exhibited a lower frequency of class 4 glomerulonephritis, had lower disease activity, and received lower CS than those who did not HCQ was associated with a longer time to the occurrence of renal damage (HR, 0.12; 95% CI, 0.02 to 0.97)
Pokroy-Shapira *et al.*^48^ 2013	Retrospective analysis of prospectively collected data	256 patients with SLE over a 25-yr period	HCQ: 89% CS: 70% CyC: 16% MMF: 4% Aza: 14% MTX: 11% RTX: 2%	75 (31%) were diagnosed with LN and 45 (18%) had a biopsy-proven LN The HR for kidney failure was significantly higher in patients with LN than without (*P* < 0.001) Earlier kidney failure was positively associated with HTN (*P* = 0.01), older age (*P* = 0.01), LN (*P* < 0.001) and negatively associated with HCQ treatment (*P* < 0.001)
Galindo-Izquierdo *et al.*^67^ 2016	Cross-sectional, multicenter study	3575 patients with SLE, of whom 1092 (31%) had LN	HCQ (78%) IS including CS, CyC, Aza, MMF, MTX, RTX	The risk for LN was higher in men (*P* < 0.001), younger patients (*P* < 0.001), and Hispanic patients (*P* = 0.03) Relapses were associated with persistent lupus activity at last visit (*P* < 0.001) and kidney failure (*P* < 0.001) Patients receiving HCQ had a significantly lower risk of developing LN (*P* < 0.001) and kidney failure (*P* < 0.001), and responded better to specific treatments for LN (*P* = 0.014)
Wu *et al.*^52^ 2020	Retrospective study	783 patients with SLE with HCQ treatment	Group 1: HCQ for <90 d Group 2: HCQ for >90 d within 1 yr	Cumulative incidence of kidney failure showed no significant differences between groups, concluding that HCQ use in SLE is neutral in the risk of kidney failure
Peña-Vizcarra *et al.*^57^ 2023	Retrospective study	424 patients with LN	Antimalarial use (82%) versus no use (17%), on top of IS (induction) CS+MMF (54%) CS+CyC (34%) CS+Aza (12%) CS+MMF+calcineurin inhibitor (2%)	Antimalarials associated with higher complete response (HR, 1.57; 95% CI, 1.08 to 2.27), lower incidence of kidney flares (HR, 0.63; 95% CI. 0.43 to 0.92) and lower progression to kidney failure (HR, 0.37; 95% CI, 0.23 to 0.53) The effect of antimalarials was modified by baseline eGFR, histological class, and/or concomitant initial IS

Aza, azathioprine; CI, confidence interval; CS, corticosteroid; CsA, cyclosporine; CyC, cyclophosphamide; HCQ, hydroxychloroquine; HR, hazard ratio; HTN, hypertension; IS, immunosuppression; LN, lupus nephritis; MMF, mycophenolate mofetil; MTX, methotrexate; RTX, rituximab; Tac, tacrolimus.

Sisó *et al*.^47^ found that patients treated with antimalarials before LN diagnosis had lower frequency of elevated serum creatinine, kidney failure, and mortality, with multivariable analyses, suggesting a borderline protective effect on kidney failure and a significant effect on survival. A retrospective study involving 256 patients showed that HCQ was associated with a lower risk of kidney failure in those with LN.^48^

The Lupus in Minorities: Nature Versus Nurture study was a longitudinal observational cohort of patients with SLE with kidney involvement. Among the different subanalyses performed, the authors evaluated HCQ's role in delaying kidney damage.^49^ Among 203 analyzed patients, 63 (31%) developed kidney damage, primarily due to proteinuria. Cox regression analysis suggested that HCQ provided a protective effect against kidney damage (hazard ratio, 0.12; 95% CI, 0.02 to 0.97; *P* = 0.046). However, these findings were controversial^50,51^ because of concerns over confounding by indication, where patients perceived as lower risk were more likely to receive HCQ, whereas higher-risk patients received more aggressive therapies. In addition, potential immortal person-time bias was noted because patients must have survived without kidney damage to receive HCQ, granting them a survival advantage. Other confounding factors included differences in nephroprotective medications, lack of data on HCQ dosage and duration of maintenance IS, and other residual biases, raising questions about the validity of the protective effect attributed to HCQ.

A retrospective population-based cohort study involving 2050 patients with newly diagnosed SLE^52^ revealed no statistical difference in kidney failure risk between patients using HCQ for ≥90 days compared with <90 days users.

## Dose, Compliance, and Long-Term Side Effects of Antimalarials

Although antimalarials are generally well tolerated, they can cause significant long-term side effects that increase health care costs.^53^ One notable complication is retinal toxicity, which, although rare (0.3%–8.8% prevalence after 7 years), can lead to irreversible maculopathy because of drug accumulation in the pigmented epithelium.^54–57^ Patients with LN and kidney impairment may be more susceptible to this risk because HCQ is excreted by the kidneys.^53^ A prevalence of 7.5% for subclinical retinopathy was found in patients taking HCQ at 6.5 mg/kg, indicating that subclinical toxicity may be more common than clinical cases.^55^ Consequently, European League for Rheumatism SLE guidelines now recommend a daily HCQ dose of ≤5 mg/kg, raising concerns about increased flare risks.^58^ A recent retrospective case-crossover study found that HCQ doses of ≤5 mg/kg are associated with a higher incidence of SLE flares.^59^

Antimalarials can cause rare but serious side effects, including cardiotoxicity (cardiomyopathy, heart failure, prolonged QT interval), particularly in patients with kidney impairment or those on high doses of HCQ.^4^ The coronavirus disease 2019 pandemic highlighted these risks.^60,61^ Other relatively common side effects include gastrointestinal manifestations, such as nausea and diarrhea, whereas cutaneous reactions, like skin rashes or hyperpigmentation, may also occur.^3^ Other rarer effects include headache, myopathy, and ototoxicity.

Finally, compliance with HCQ is a concern, as nonadherence can exceed 50%.^6,62,63^

Taken together, given the low-quality evidence and conflicting results regarding their benefits for LN, with the risk of rare but serious adverse effects could be an additional reason to individualize their use.^63^

## Conclusions

The evidence on antimalarials for disease remission, flare prevention, or kidney failure in patients with LN is inadequate. Most studies are observational with small sample sizes conducted under outdated management practices. The rationale for discontinuing IS therapy is unclear because many patients with active disease are at high risk of relapse. The potential renal benefits of HCQ in patients who achieve both clinical and immunologic remission off IS remain unverified. Nonadherence to HCQ further questions the reliability of retrospective analyses. In addition, recent findings suggest that commonly prescribed HCQ doses (*e*.*g*., 5 mg/kg per day or lower) may reduce its efficacy.^58^ Therefore, the current dosing guidelines likely do not provide the anticipated benefits.

**Figure 1:**
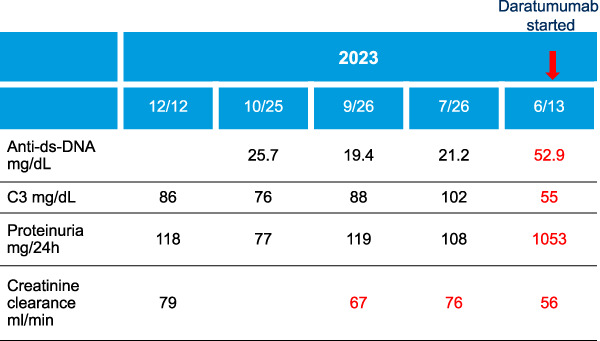
**Twenty-five-year-old female patient with SLE originally diagnosed in 2020 after presenting with skin rash, joint pain, dry eyes, and serology positive for ANA, anti-dsDNA, anti-Smith, and several other serologies.** She was initiated on prednisone and Aza. Aza dose was increased to 200 mg daily in November 2021. In May 2023, she was found to have active urinary sediment, SCr had increased to 1.1 mg/dl, and anti-dsDNA antibodies were positive, with low complement levels. She underwent a kidney biopsy in May 2023 that showed a focal proliferative and membranous LN (International Society of Nephrology/Renal Pathology Society class 3 and 5). She was started on prednisone 60 mg daily with a taper together with MMF 1000 mg twice daily. When seen at Mayo Clinic in June 2023, given the absence of significant response to the combination therapy she was enrolled in the phase 2 open-label trial evaluating the efficacy and safety of daratumumab in treatment of patients with active LN (NCT04868838). She received daratumumab 1800 mg S.C. once weekly for 8 weeks and then once every 2 weeks for eight additional doses. Prednisone was discontinued by mid October 2023, and she was continued on MMF 1000 mg twice a day. She received no HCQ as part of the induction treatment protocol. The patient went into complete immunological (negative anti-dsDNA) and clinical remission (normal C3/C4 complement levels) with proteinuria 118 mg/24 hours. ANA, anti-nuclear antibody; Anti-dsDNA (ELISA) ref. range <30 UI/ml; C3 ref. range 75–175 mg/dl. Anti-dsDNA, anti-double stranded DNA antibodies; Aza, azathioprine; HCQ, hydroxychloroquine; LN, lupus nephritis; MMF, mycophenolate mofetil; S.C., sub-cutaneous; SCr, serum creatinine.

In the current era of individualized and targeted therapies, a one-size-fits-all approach to treatment is increasingly outdated. The evidence reviewed raises questions about whether the therapeutic benefits of HCQ in LN are more perception-based than clinically supported. Contemporary targeted therapies for LN, such as anti-CD20 monoclonal antibodies (rituximab, obinutuzumab), anti-CD38 monoclonal antibodies (daratumumab), B-cell maturation antigen bispecific antibodies (teclistamab), and CAR-T therapy, offer more tailored and effective options. To believe that adding HCQ on top of such modern therapies for remission induction would make a difference no longer makes sense. The case presented in Figure 1 illustrates this point. Consequently, it is imperative to shift toward more evidence-based and individualized treatments approach. Prospective trials to accurately define the role of antimalarials in LN are needed.

## Data Availability

The data underlying this article will be shared on reasonable request to the corresponding author.
